# Pile-fermentation and golden-flower fermentation reshape tea polysaccharides in Tibetan dark tea: structural characteristics and lipid-modulating effects in *Caenorhabditis elegans*

**DOI:** 10.1016/j.fochx.2026.104390

**Published:** 2026-08-31

**Authors:** Manyou Yu, Ana Novo Barros, Irene Gouvinhas, Junlin Deng, Zhuoya Xiang, Yinghao Yuan, Ting Zhang, Xiaobo Tang, Chen Xia, Shengting Yang

**Affiliations:** a Institute of Agro-products Processing Science and Technology/Institute of Food Nutrition and Health, Sichuan Academy of Agricultural Sciences (SAAS), 610066 Chengdu, China; b Centre for the Research and Technology of Agro-Environmental and Biological Sciences/Institute for Innovation, Capacity Building and Sustainability of Agri-Food Production (CITAB/Inov4Agro), University of Trás-os-Montes and Alto Douro (UTAD), 5000-801 Vila Real, Portugal; c Animal and Veterinary Research Center (CECAV), University of Trás-os-Montes and Alto Douro, Vila Real 5000-801, Portugal; d Tea Research Institute, Sichuan Academy of Agricultural Sciences, 610066 Chengdu, China; e Sichuan Academy of Agricultural Sciences, 610066 Chengdu, China

**Keywords:** Tibetan dark tea, Tea polysaccharides, Pile-fermentation, Golden-flower fermentation, Structural characteristics, Lipid-modulating activity

## Abstract

Pile-fermentation and golden-flower fermentation are key processing steps in Tibetan dark tea, yet their combined effects on tea polysaccharides remain poorly understood. To distinguish the individual and combined contributions of pile-fermentation and golden-flower fermentation, four polysaccharide-enriched fractions were prepared in a 2 × 2 design: LTPS (neither process), LFTPS (golden-flower fermentation only), HTPS (pile-fermentation only), and HFTPS (both processes applied sequentially). All fractions were identified as uronic acid-rich acidic heteropolysaccharides with similar monosaccharide species but distinct molar ratios. Processing markedly altered the molecular-weight distribution, and HFTPS showed the lowest apparent molecular weight and the highest absolute zeta potential. UV–Vis, FT-IR, XRD, and SEM analyses indicated that the main polysaccharide backbone remained largely preserved, whereas microstructural and physicochemical properties were substantially reshaped by processing. Among the four fractions, HFTPS exhibited the strongest antioxidant capacity and the highest inhibitory activities against α-glucosidase, cholesterol esterase, and lipoxygenase. In a high-glucose-induced *Caenorhabditis elegans* model, tea polysaccharides reduced lipid accumulation, improved locomotion, prolonged lifespan, and enhanced heat- and oxidative-stress resistance. These phenotypic improvements were accompanied by biochemical changes and transcriptional responses consistent with enhanced fatty acid oxidation and reduced lipogenesis. Exploratory correlation analysis further suggested that lower apparent molecular weight, higher polyphenol content, and more pronounced acidic and composite characteristics were generally associated with improved bioactivities. Overall, the fraction obtained after pile-fermentation followed by golden-flower fermentation showed the most favourable integrated structural and functional profile among the four fractions, supporting the potential use of Tibetan dark tea polysaccharides in functional food applications.

## Highlights

• Pile-fermentation markedly reduced the apparent molecular weight of tea polysaccharides.
• HFTPS combined the lowest apparent molecular weight with the strongest overall bioactivities.
• HFTPS reduced lipid accumulation and improved stress resistance in *C. elegans*.
• Processing-associated structural variation was linked to antioxidant and enzyme-inhibitory activity.
• Transcriptional responses were consistent with enhanced fatty acid oxidation and reduced lipogenesis.

## 1 Introduction

Tea is one of the most widely consumed non-alcoholic beverages worldwide and is valued for both its sensory qualities and health-promoting potential (Zhang et al., 2024b). Among the six major tea categories in China, dark tea is distinguished by its post-fermentation process, during which intense microbial activity and chemical transformation reshape the composition and functionality of tea constituents (Chen et al., 2025b; Guo et al., 2025; Liu et al., 2025; Ma et al., 2026). Compared with non-post-fermented teas, dark tea undergoes more extensive polyphenol oxidation and polymerisation, carbohydrate rearrangement, and interactions among multiple components, resulting in characteristic flavour profiles and distinctive bioactive properties. Tibetan dark tea, an important type of dark tea mainly produced in Ya'an, Sichuan, often undergoes pile-fermentation, ageing, and, in some products, an additional “golden-flower fermentation” step dominated by *Eurotium cristatum* (Chen et al., 2025a; Sun et al., 2023; Xu et al., 2025). These processing stages are considered crucial for product quality formation, yet the specific effects of pile-fermentation and golden-flower fermentation on individual bioactive fractions remain insufficiently understood (Chen et al., 2025a; Sun et al., 2023).

Tea polysaccharides are among the major functional constituents of tea. Their physicochemical properties and biological activities are closely associated with molecular weight distribution, monosaccharide composition, acidic-group abundance, chain conformation, and interactions with *co*-existing compounds such as proteins and polyphenols (Huang & Yu, 2024; Qi et al., 2026; Walayat et al., 2025). Previous studies have suggested that fermentation-related microorganisms may secrete enzymes capable of promoting depolymerisation, debranching, and charge-related modification of polysaccharide systems, thereby altering solubility, aggregation behaviour, and interfacial properties, and ultimately affecting antioxidant and enzyme-inhibitory functions (Liu et al., 2024; Wang et al., 2025b; Yang et al., 2025). In particular, golden-flower fermentation introduces a dominant fungal community and is frequently accompanied by nutritional supplementation that supports fungal growth and metabolic activity (Wang et al., 2025b). However, under a controlled raw-material system, the individual and combined effects of pile-fermentation and golden-flower fermentation on tea polysaccharides—especially with respect to molecular weight, acidic sugar proportion, and co-associated components—have not yet been systematically clarified.

Obesity and related metabolic disorders are major global health concerns and are closely associated with type 2 diabetes, non-alcoholic fatty liver disease, and cardiovascular disease (Abad-Jiménez & Vezza, 2025; Fang et al., 2025). In this context, food-derived polysaccharides have attracted increasing attention because they may regulate lipid metabolism through multiple routes, including antioxidant protection, inhibition of digestive and metabolism-related enzymes, and modulation of metabolic signalling pathways (Fang et al., 2025; Zhou et al., 2025). Tea polysaccharides generated during the fermentation of Tibetan dark tea may therefore possess differentiated lipid-modulating potential owing to their unique structural and compositional characteristics (Fang et al., 2025; Peng et al., 2025; Zhou et al., 2025). Nevertheless, most existing studies remain descriptive, focus on a single tea sample or a single process, and rarely provide a systematic comparison across the full pile-fermentation–flowering processing chain. As a result, the links between structural variation and lipid-related phenotypes remain poorly defined.

In the present study, we hypothesised that pile-fermentation and golden-flower fermentation jointly influence the molecular-weight profile, acidic characteristics, and aggregation behaviour of Tibetan dark tea polysaccharides through microbially driven structural modification and interactions with accompanying components. These changes may further translate into differences in antioxidant performance, inhibition of lipid metabolism-related enzymes, and regulation of lipid accumulation *in vivo*. To test this hypothesis, a 2 × 2 processing design was established to generate four tea polysaccharide fractions with or without pile-fermentation and with or without golden-flower fermentation. Their basic composition, molecular-weight distribution, monosaccharide composition, and physicochemical characteristics were systematically characterised, followed by *in vitro* functional evaluation and *in vivo* validation using a high-glucose-induced *Caenorhabditis elegans* model. This work aims to provide a process-oriented and structure-informed basis for the functional development of Tibetan dark tea polysaccharides.

## 2 Materials and methods

### 2.1 Materials and reagents

**Fig. 1:**
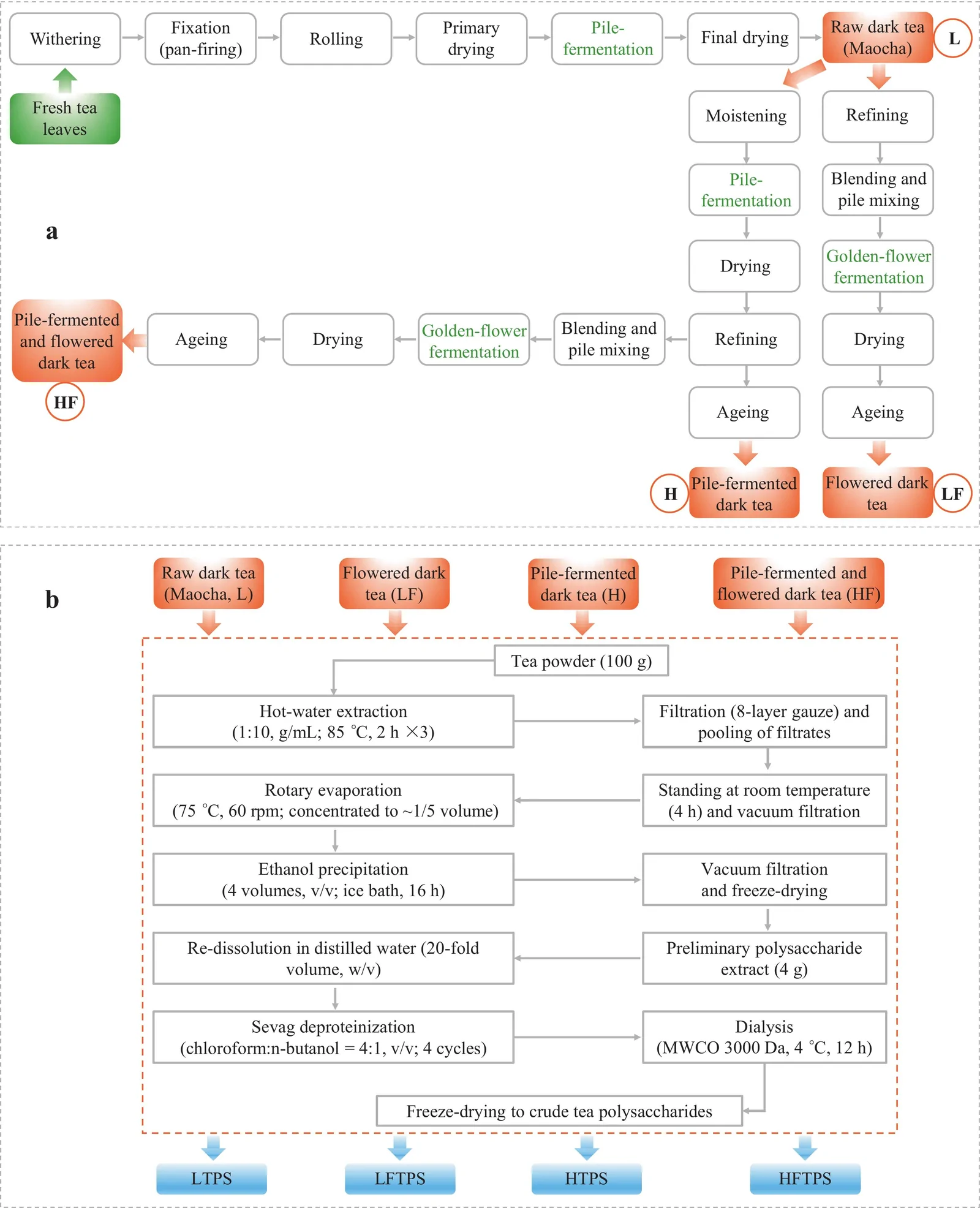
Processing strategies of four dark teas and extraction procedure of crude polysaccharides. (a) Processing schemes of four dark teas with different fermentation strategies, including raw dark tea (Maocha, L), flowered dark tea (LF), pile-fermented dark tea (H), and pile-fermented and flowered dark tea (HF). Key fermentation steps are highlighted in green. These distinct processing pathways generated tea samples with different polysaccharide structures and bioactivities. (b) Extraction procedure for crude polysaccharides from the four dark teas. Tea samples were extracted with hot water, followed by ethanol precipitation, Sevag deproteinization, dialysis, and freeze-drying. The resulting crude polysaccharide fractions were designated as LTPS, LFTPS, HTPS, and HFTPS according to their tea sources. (For interpretation of the references to colour in this figure legend, the reader is referred to the web version of this article.)

Four tea materials, including raw dark tea (mao-cha), flowered mao-cha, pile-fermented mao-cha, and pile-fermented plus flowered mao-cha, were provided by Ya'an Yazhou Hengtai Tea Industry Co., Ltd. (Ya'an, Sichuan, China). Fig. 1a showed the processing schemes of the four dark teas with different fermentation strategies. The wild-type *Caenorhabditis elegans* strain N2 and its feeding bacterium uracil-auxotrophic *Escherichia coli* OP50 were kindly supplied by Sichuan Normal University.

Analytical-grade anhydrous ethanol, chloroform, and calcium carbonate were purchased from Chengdu Kelon Chemical Reagent Factory (Chengdu, China). Phenol (analytical reagent grade), sulphuric acid (95–98%, *w*/w, analytical reagent grade), Folin–Ciocalteu reagent, and hydrochloric acid (36–38%, *w*/w, analytical reagent grade) were obtained from Chengdu Kelong Chemicals Co., Ltd. (Chengdu, China). Sodium hypochlorite was purchased from Tianjin Jindong Tianzheng Fine Chemical Reagent Factory (Tianjin, China). Peptone, agar, sodium chloride, cholesterol, glucose, magnesium sulphate, and calcium chloride were purchased from Beijing Aoboxing Biotechnology Co., Ltd. (Beijing, China).

Coomassie Brilliant Blue, bovine serum albumin (BSA) standard, glucose standard, galacturonic acid standard, α-glucosidase, *p*-nitrophenyl-α-D-glucopyranoside (pNPG), and monosaccharide standards including D-mannose (D-Man), L-rhamnose (L-Rha), D-glucuronic acid (D-GlcA), D-galacturonic acid (D-GalA), d-glucose (D-Glu), D-xylose (D-Xyl), D-galactose (D-Gal), L-arabinose (L-Ara), D-fucose (D-Fuc), 1,1-diphenyl-2-picrylhydrazyl (DPPH), 2,2′-azinobis-(3-ethylbenzothiazoline-6-sulfonic acid) (ABTS), Trolox, a total antioxidant capacity (T-AOC) assay kit, acarbose, orlistat, cholesterol esterase, sodium taurocholate, *p*-nitrophenyl butyrate (PNPB), and Oil Red O were purchased from Shanghai Yuanye Bio-Technology Co., Ltd. (Shanghai, China). A lipoxygenase inhibitor screening kit, a bicinchoninic acid (BCA) protein assay kit, and assay kits for superoxide dismutase (SOD), catalase (CAT), non-esterified fatty acids (NEFA), malondialdehyde (MDA), and triglycerides (TG) were obtained from Nanjing Jiancheng Bioengineering Institute (Nanjing, China). TRIzol reagent was obtained from Sangon Biotech (Shanghai) Co., Ltd. (Shanghai, China). HiScript III All-in-one RT SuperMix and Taq Pro Universal SYBR Vazyme qPCR Master Mix were purchased from Vazyme Biotech Co., Ltd. (Nanjing, China). Unless otherwise specified, small-molecule chemicals were of analytical reagent grade, chromatographic standards were of HPLC grade, and biochemical kits and reagents were of assay grade as supplied by the manufacturers.

### 2.2 Preparation of polysaccharides from Tibetan dark tea

For each tea material, 100 g of dried sample was extracted with distilled water at a solid-to-liquid ratio of 1:10 (g/mL) in a water bath at 85 °C for 2 h (Fig. 1b). The extract was filtered through eight layers of gauze. This extraction was repeated three times, and the filtrates were combined. After standing at room temperature for 4 h, the supernatant was collected by vacuum filtration. The filtrate was concentrated by rotary evaporation at 75 °C (60 rpm) to approximately one-fifth of the initial volume. Polysaccharides were precipitated by adding four volumes of anhydrous ethanol, mixing thoroughly, and incubating in an ice bath for 16 h. The precipitate was collected by vacuum filtration and freeze-dried to yield a preliminary polysaccharide extract.

The dried extract (4 g) was re-dissolved in distilled water (20-fold volume, *w*/*v*). Proteins were removed using the Sevag method (chloroform:*n*-butanol = 4:1, *v*/v) for four cycles. The aqueous phase was transferred into a dialysis bag (MWCO 3.0 kDa) and dialysed against distilled water at 4 °C for 12 h. The retentate was then freeze-dried to obtain crude polysaccharides. Polysaccharides from raw mao-cha, flowered mao-cha, pile-fermented mao-cha, and pile-fermented plus flowered mao-cha were designated as LTPS, LFTPS, HTPS, and HFTPS, respectively.

### 2.3 Basic chemical composition of tea polysaccharides

The basic composition of tea polysaccharides included neutral sugars, uronic acids, polyphenols, proteins, and amino acids. Unless otherwise stated, all measurements were performed in triplicate. Results were expressed on a dry-weight basis as g/100 g or mg/g and are reported as mean ± standard deviation.

#### 2.3.1 Neutral sugar content

Neutral sugars were determined by the phenol–sulphuric acid method (DuBois et al., 1956). Glucose was used to prepare standard solutions for calibration. An appropriate volume of sample solution was mixed with 5% (*w*/*v*) phenol, followed by rapid addition of 98% (*w*/w) sulphuric acid. After colour development at room temperature, absorbance was measured at 490 nm. Neutral sugar content was calculated from the glucose standard curve using the reagent blank as control.

#### 2.3.2 Uronic acid content

Uronic acids were quantified using the *m*-hydroxydiphenyl method (Xia et al., 2025). D-galacturonic acid was used to prepare the standard curve. Sample solutions were reacted with the sulphuric acid–borate system, followed by colour development with *m*-hydroxydiphenyl. Absorbance was measured at 520 nm, and uronic acid content was calculated from the standard curve after blank correction.

#### 2.3.3 Polyphenol content

Total polyphenols were measured using the Folin–Ciocalteu assay (Yu et al., 2024). Gallic acid was used as the calibration standard. Briefly, sample solutions were mixed with Folin–Ciocalteu reagent and then neutralised with sodium carbonate. After incubation in the dark, absorbance was measured at 765 nm. Results were expressed as mg gallic acid equivalents (GAE)/g dry sample. Sample blanks were included to minimise interference from inherent sample colour.

#### 2.3.4 Protein content

Protein content was determined by the Bradford method using Coomassie Brilliant Blue (Yu et al., 2024). Bovine serum albumin (BSA) was used to establish the standard curve. After reaction with Bradford reagent, absorbance was measured at 595 nm, and protein content was calculated using the reagent blank as control.

#### 2.3.5 Amino acid content

Amino acid composition was determined after acid hydrolysis followed by pre-column derivatisation and HPLC analysis. Briefly, a homogenised sample (0.200 g) was placed in a sealed hydrolysis vial, and 5.0 mL of 6 mol/L HCl containing 1% phenol was added. The vial was flushed with nitrogen for 1 min, sealed, and hydrolysed at 110 °C for 22 h. After cooling, the hydrolysate was quantitatively transferred and diluted to 50 mL with ultrapure water. An aliquot (1.0 mL) was evaporated to dryness under nitrogen at 95 °C to remove residual HCl. The residue was re-dissolved in 1.0 mL of 0.01 mol/L HCl, mixed thoroughly, and filtered through a 0.22 μm membrane.

Amino acids were then determined using an online pre-column derivatisation method. Primary amino acids were derivatised with *O*-phthalaldehyde (OPA), and secondary amino acids were derivatised with 9-fluorenylmethyl chloroformate (FMOC). Separation was performed on a ZORBAX Eclipse AAA column (4.6 × 150 mm, 3.5 μm). Detection was carried out at 338 nm for 0–19 min and at 266 nm for 19.01–25 min. Mobile phase A was 40 mM sodium phosphate buffer (pH 7.8), and mobile phase B was acetonitrile/methanol/water (45,45,10, *v*/v/v). The flow rate was 1.0 mL/min, and the column temperature was maintained at 45 °C. The elution programme was as follows: 0–1 min, 100% A; 23 min, 43% A/57% B; 27–34 min, 0% A/100% B; 40–41 min, 100% A. Amino acids were quantified by external standard calibration and expressed as mg/g dry sample.

### 2.4 Structural characterization of polysaccharides

#### 2.4.1 Monosaccharide composition analysis

Monosaccharide composition and molar ratios were analysed by PMP (1-phenyl-3-methyl-5-pyrazolone) derivatisation coupled with HPLC (Xia et al., 2025). Briefly, 20 mg of polysaccharide sample was hydrolysed with 4 mL of 2 mol/L H₂SO₄ at 105 °C for 8 h in a sealed ampoule. After cooling, BaCO₃ was added to adjust the pH to 7.0, and the resulting BaSO₄ precipitate was removed by centrifugation. The precipitate was washed with deionised water, and the supernatants were combined, filtered, and concentrated to 0.5 mL. The concentrate was reacted with 200 μL of 0.5 mol/L PMP in methanol and 400 μL of 0.3 mol/L NaOH at 70 °C for 40 min. After neutralisation with HCl, excess PMP and by-products were removed by three sequential extractions with chloroform, using a chloroform-to-aqueous-phase ratio of 1:1 (*v*/v) in each extraction. The aqueous phase was used for HPLC analysis.

HPLC was performed on a ZORBAX Eclipse XDB-C18 reversed-phase column (150 mm × 4.6 mm, 5 μm; Agilent Technologies, Santa Clara, CA, USA). The mobile phase consisted of acetonitrile and phosphate buffer (pH 6.9) with gradient elution (0–30 min, acetonitrile 8–20%). The flow rate was 1.0 mL/min, the detection wavelength was 250 nm, and the column temperature was 30 °C. Results were expressed as molar percentage (mol%).

#### 2.4.2 Molecular weight distribution (GPC)

Molecular weight distribution was determined by gel permeation chromatography (GPC) (Yu et al., 2024). A series of dextran standards was used to establish the calibration curve, yielding the regression equation y = −0.549× + 10.003 (R^2^ = 0.9942). Polysaccharide solutions were filtered before injection, and apparent molecular weight parameters were calculated based on the calibration curve.

#### 2.4.3 UV–vis scanning

Polysaccharide solutions (0.1 mg/mL) were scanned over 200–400 nm using a UV–Vis spectrophotometer (UV-3600) to evaluate characteristic absorption associated with *co*-extracted components such as proteins and polyphenols.

#### 2.4.4 Zeta potential measurement

The zeta potential of aqueous polysaccharide solutions was measured using a particle size and zeta potential analyser (Zetasizer Nano). Samples were prepared at the same concentration, and each sample was measured at least three times. Results are reported as mean ± standard deviation.

#### 2.4.5 Thermogravimetric analysis (TGA)

Thermogravimetric analysis was performed using 20.0 mg of polysaccharide powder. Samples were heated from 25 to 800 °C at a rate of 10 °C/min under a nitrogen atmosphere to evaluate thermal stability and decomposition behaviour.

#### 2.4.6 Fourier-transform infrared spectroscopy (FT-IR)

Polysaccharide samples were mixed thoroughly with KBr, ground, and pressed into pellets. FT-IR spectra were recorded on a Thermo IS 50 spectrometer over 1000–4000 cm^−1^ with 32 scans per sample to characterise functional groups.

#### 2.4.7 X-ray diffraction (XRD)

Powder samples were evenly spread on the sample holder and analysed using an X-ray diffractometer (Rigaku Ultima IV) operated at 40 kV and 40 mA. Diffraction patterns were collected over a 2θ range of 5–90° to evaluate crystalline and amorphous characteristics.

#### 2.4.8 Scanning electron microscopy (SEM)

Polysaccharide powders were mounted on conductive adhesive tape, sputter-coated with gold under vacuum, and observed using a scanning electron microscope (Zeiss Sigma300). Imaging was performed at an accelerating voltage of 10 kV and a working distance of 5.9 mm.

### 2.5 *In vitro* antioxidant activity and inhibition of key metabolic enzymes

The *in vitro* antioxidant activity of tea polysaccharides was evaluated by ABTS and DPPH radical scavenging assays and ferric reducing antioxidant power (FRAP) (Xia et al., 2025). For the ABTS assay, 15 μL of sample solution at different concentrations was mixed with 185 μL of ABTS working solution and incubated in the dark at room temperature for 30 min. Absorbance was measured at 734 nm. Trolox was used as the positive control and distilled water as the blank control. For the DPPH assay, 10 μL of sample solution was mixed with 190 μL of DPPH working solution and incubated in the dark at room temperature for 30 min, followed by absorbance measurement at 520 nm. FRAP was determined according to the manufacturer's instructions and expressed as Fe^2+^-reducing capacity.

Enzyme inhibitory activities were evaluated against α-glucosidase, lipoxygenase, and cholesterol esterase. For α-glucosidase inhibition, 20 μL of sample solution was mixed with 50 μL of α-glucosidase (1 U/mL) and pre-incubated at 37 °C for 10 min. Then, 50 μL of pNPG (5 mmol/L) was added and the mixture was incubated at 37 °C for 30 min. The reaction was terminated by adding 50 μL of Na₂CO₃ (0.2 mol/L), and absorbance was recorded at 405 nm. Acarbose was used as the positive control.

Lipoxygenase inhibitory activity was measured using a commercial screening kit according to the manufacturer's instructions. Nordihydroguaiaretic acid (NDGA) was used as the positive control. For cholesterol esterase inhibition, 50 μL of sample solution was mixed with 50 μL of cholesterol esterase (1 U/mL) and pre-incubated at 37 °C for 10 min. Subsequently, 100 μL of PNPB (20 μmol/L) was added and the reaction was allowed to proceed at 37 °C for 15 min. Absorbance was measured at 405 nm. Orlistat was used as the positive control. For all three enzyme assays, the mean inhibition rate at each tested concentration was first calculated from three replicates. The half-maximal inhibitory concentration (IC₅₀) was then determined by linear interpolation between the two adjacent tested concentrations bracketing 50% inhibition.

### 2.6 *C. elegans* culture and experimental design

#### 2.6.1 Preparation of media

Standard NGM plates were prepared as follows (200 mL) (Stiernagle, 2006): peptone (0.5 g), NaCl (0.6 g), and agar (3.6 g) were dissolved in 195 mL of water and sterilised by autoclaving at 121 °C for 20 min. After cooling to approximately 55 °C, sterile phosphate buffer (pH 6.0, 5 mL), MgSO₄ (1 mol/L, 0.2 mL), CaCl₂ (1 mol/L, 0.2 mL), and cholesterol in ethanol (5 mg/mL, 0.2 mL) were added.

High-glucose NGM plates were prepared to a final glucose concentration of 100 mmol/L. Glucose (3.6 g) was dissolved in water to 20 mL and sterilised separately at 115 °C for 30 min. The glucose solution was then mixed with the NGM base medium and poured into plates.

#### 2.6.2 Culture of *E. coli* OP50

Frozen *E. coli* OP50 stock was streaked on LB agar and incubated at 37 °C to obtain single colonies. A single colony was inoculated into LB broth and cultured at 37 °C with shaking at 180 rpm for 12–18 h until the OD₆₀₀ reached 1.0. The culture was used as the food source for worms.

#### 2.6.3 Synchronisation of *C. elegans*

When plates contained abundant gravid adults and eggs, worms and eggs were washed off with M9 buffer and collected into sterile microcentrifuge tubes. After centrifugation at 4000 rpm for 2 min, the supernatant was removed and the pellet was resuspended in freshly prepared bleaching solution (ddH₂O:sodium hypochlorite:NaOH = 3.5:1:0.5, *v*/v/v). After gentle agitation for 3–5 min until adult carcasses were lysed, samples were centrifuged again and washed at least three times with M9 buffer. The final egg-containing suspension was transferred onto fresh NGM plates to obtain synchronised worms (Stiernagle, 2006).

#### 2.6.4 Experimental groups

Synchronised L1 larvae were assigned to seven groups: normal control (NC), maintained on standard NGM plates; model control (CM), maintained on high-glucose NGM plates; positive control (OST), maintained on high-glucose NGM plates supplemented with orlistat (50 μg/mL); and four polysaccharide-treated groups maintained on high-glucose NGM plates supplemented with LTPS, LFTPS, HTPS, or HFTPS (each 500 μg/mL), designated as L, LF, H, and HF, respectively. All groups were cultured on plates seeded with *E. coli* OP50. Except for the NC group, all other groups were exposed to high-glucose medium.

### 2.7 Phenotypic, lifespan, stress-resistance and lipid-staining assays in *C. elegans*

#### 2.7.1 Body size and locomotion assays

Synchronised worms were transferred to the corresponding treatment plates and cultured for 3 d. Worms were then transferred onto unseeded plates and imaged under a fluorescence microscope. Body length and body width were quantified using ImageJ software, with 10 worms analysed per group.

For locomotion assays, worms were cultured for 3 d under the designated treatments. For head-thrashing measurement, an individual worm was transferred to an unseeded plate with a drop of M9 buffer, and the number of head thrashes within 1 min was counted. For body bending, worms were placed on unseeded plates and the number of body bends within 1 min was recorded. One body bend was defined as one complete sinusoidal movement.

#### 2.7.2 Lifespan assay

Synchronised worms were transferred to treatment plates as described above. Each group contained 30 worms per plate with three independent replicate plates. The day of transfer was defined as day 0. Worms were transferred to fresh plates every 24 h. Survival status, including live, dead, and missing worms, was recorded daily until all worms had died.

#### 2.7.3 Heat- and oxidative-stress survival assays

For the heat-stress assay, worms were cultured for 3 d under the corresponding treatment conditions and then transferred to fresh plates of the same treatment. Each group included 30 worms per plate with three independent replicate plates. Plates were incubated at 35 °C, and survival was monitored every 1 h until all worms had died. Death was defined as the absence of movement and pharyngeal pumping after gentle stimulation. Worms that crawled off the plate, were mechanically damaged, or were lost during transfer were treated as censored observations.

For oxidative-stress survival, NGM medium was cooled to 50 °C and supplemented with H₂O₂ to a final concentration of 0.3% (*v*/v). The medium was mixed, poured into plates, and allowed to solidify in the dark. To avoid bacterial interference, no OP50 was added to oxidative-stress plates. Age-matched worms from each treatment were transferred onto oxidative-stress plates (30 worms per plate, three replicate plates) and incubated at 20 °C. Survival was assessed every 0.5 h until all worms had died. Death and censoring criteria followed those used in the heat-stress assay.

#### 2.7.4 Oil red O staining and quantification

After 3 d of treatment, worms were collected and washed three times with M9 buffer to remove surface bacteria (An et al., 2023). Worms were fixed with 1.0 mL M9 buffer containing 50 μL of 10% formaldehyde, followed by three freeze–thaw cycles (−80 °C for 20 min and thawing at room temperature; the final thaw was performed slowly on ice). After fixation, worms were washed three times with pre-cooled M9 buffer.

Worms were dehydrated with 100% propylene glycol for 5 min and stained with 1.5 mL of 0.5% (*w*/*v*) filtered Oil Red O solution at 37 °C with shaking at 220 rpm for 4 h. Samples were then differentiated sequentially with 98% and 85% propylene glycol for 5 min each and washed twice with M9 buffer. Worms were mounted on 2% agarose pads and imaged under identical exposure conditions. Oil Red O-positive signals were quantified using ImageJ as the percentage of red-stained area relative to total worm area. At least 10 worms were analysed per group.

### 2.8 Determination of enzyme activities and metabolic indices in *C. elegans*

Synchronised L1 larvae were cultured under the corresponding treatment conditions for 3 d. Worms were collected and washed three times with M9 buffer to remove residual OP50, and pellets were resuspended in pre-cooled lysis buffer or PBS. Samples were homogenised thoroughly and centrifuged at 4 °C (approximately 12,000 rpm for 10 min), and the supernatants were collected for subsequent analyses.

Total protein concentration was determined using the BCA method. Superoxide dismutase (SOD) and catalase (CAT) activities, as well as triglycerides (TG), non-esterified fatty acids (NEFA), and malondialdehyde (MDA), were quantified using the corresponding commercial kits according to the manufacturers' instructions. All results were normalized to total protein content and expressed as activity or content per mg protein. Each group included at least three independent biological replicates.

### 2.9 Quantification of lipid metabolism-related gene expression by RT–qPCR

Synchronised L1 larvae were cultured under the designated treatment conditions for 3 d. Worms were collected, washed three times with M9 buffer, and pelleted. Total RNA was extracted using TRIzol reagent after thorough homogenisation. RNA concentration and purity were assessed spectrophotometrically using the A260/A280 ratio. Qualified RNA was reverse-transcribed into cDNA using HiScript III All-in-one RT SuperMix according to the manufacturer's instructions.

Quantitative PCR was performed using Taq Pro Universal SYBR Vazyme qPCR Master Mix. Target gene sequences were obtained from the NCBI database, and primers were designed using Primer5 software; primer sequences are listed in **Table S1**. The cycling programme was 95 °C for 30 s, followed by 40 cycles of 95 °C for 10 s and 60 °C for 30 s. A melting-curve analysis from 65 to 95 °C was performed to confirm amplification specificity. The reference gene was *act-1*, and relative expression levels were calculated using the 2^−ΔΔCt method (Livak & Schmittgen, 2001). Each group included at least three independent biological replicates, and each sample was analysed in technical triplicate.

### 2.10 Statistical analysis

All data are presented as mean ± standard deviation (SD). Unless otherwise stated, each experiment included at least three independent biological replicates. Statistical analyses were performed using IBM SPSS Statistics 27 (IBM, Armonk, NY, USA) and GraphPad Prism 8 (GraphPad Software, San Diego, CA, USA). Figures were prepared using Origin 2022 and GraphPad Prism 8.

Data from the 2 × 2 processing design were analysed by two-way ANOVA to test the main effects of pile-fermentation and golden-flower fermentation, as well as their interaction. When significant effects were detected, Sidak's multiple comparisons test was used for *post hoc* comparisons. Survival data were analysed using Kaplan–Meier curves and the log-rank test. Worms lost during handling or those that crawled off the plates were treated as censored observations.

Exploratory correlation analysis among representative physicochemical properties, *in vitro* bioactivities, *in vivo* phenotypes, and pathway-level gene response indices was primarily performed using Spearman rank correlation because of the limited sample number and mixed variable types. Pearson correlation analysis was additionally performed as a supplementary sensitivity analysis. Correlation heatmaps were generated in Origin 2022.

## 3 Results and discussion

### 3.1 Polysaccharide yield and basic chemical composition

The yields and basic chemical composition of the four tea polysaccharide fractions are summarised in Table 1. Both pile-fermentation and golden-flower fermentation affected polysaccharide recovery. Compared with LTPS, the yield increased from 2.07% to 4.77% after golden-flower fermentation (LFTPS), whereas pile-fermentation produced a more pronounced increase, reaching 7.02% in HTPS. HFTPS showed a similarly high yield (6.68%), indicating that the major increase in extractability had already occurred during pile-fermentation. Increased extractable polysaccharide yield after fermentation has been reported for tea and other plant matrices, probably because microbial and endogenous enzymatic activities loosen cell-wall architecture and facilitate the release of carbohydrate-rich fractions (Huang & Yu, 2024; Wu et al., 2026).

**Table 1:** Physicochemical properties of four dark tea polysaccharides.

Properties	LTPS	LFTPS	HTPS	HFTPS
Yield (%)	2.07 ± 0.18^c^	4.77 ± 0.20^b^	7.02 ± 0.27^a^	6.68 ± 0.28^a^
Neutral sugar (%)	30.87 ± 1.67^b^	32.95 ± 1.25^ab^	32.53 ± 1.04^ab^	34.12 ± 0.88^a^
Uronic acid (%)	39.51 ± 1.38^b^	41.57 ± 1.43^ab^	40.78 ± 0.73^ab^	43.00 ± 1.26^a^
Polyphenol (%)	10.66 ± 0.01^c^	9.96 ± 0.44^d^	13.79 ± 0.17^b^	15.41 ± 0.18^a^
Protein (%)	5.25 ± 0.24^b^	4.68 ± 0.24^c^	4.38 ± 0.19^c^	5.98 ± 0.20^a^

Amino acid composition after acid hydrolysis (mg/g)				
Asp	5.55 ± 0.02^b^	5.23 ± 0.04^c^	5.61 ± 0.03^b^	7.63 ± 0.05^a^
Glu	14.22 ± 0.05^a^	11.34 ± 0.11^c^	9.94 ± 0.15^d^	12.76 ± 0.25^b^
Ser	2.76 ± 0.01^d^	3.08 ± 0.02^b^	2.92 ± 0.01^c^	4.20 ± 0.10^a^
His	0.82 ± 0.01^a^	0.54 ± 0.02^d^	0.68 ± 0.02^c^	0.77 ± 0.03^b^
Gly	4.74 ± 0.07^b^	3.93 ± 0.07^c^	3.58 ± 0.01^d^	5.08 ± 0.20^a^
Thr	1.90 ± 0.00^d^	2.29 ± 0.14^c^	2.57 ± 0.04^b^	3.85 ± 0.28^a^
Arg	1.79 ± 0.02^b^	1.39 ± 0.02^d^	1.66 ± 0.01^c^	2.23 ± 0.00^a^
Ala	5.44 ± 0.04^b^	5.38 ± 0.04^b^	4.68 ± 0.02^c^	6.53 ± 0.28^a^
Tyr	1.63 ± 0.12^c^	1.95 ± 0.16^b^	1.67 ± 0.01^c^	2.57 ± 0.36^a^
Cys	0.94 ± 0.01^a^	0.62 ± 0.03^b^	0.26 ± 0.02^d^	0.49 ± 0.03^c^
Val	1.71 ± 0.04^c^	1.83 ± 0.01^b^	1.87 ± 0.01^b^	2.73 ± 0.00^a^
Met	0.00 ± 0.00^c^	0.00 ± 0.00^c^	0.39 ± 0.00^b^	0.60 ± 0.01^a^
Phe	0.78 ± 0.07^c^	1.02 ± 0.04^b^	1.09 ± 0.02^b^	1.67 ± 0.04^a^
Ile	4.01 ± 0.19^b^	3.96 ± 0.23^c^	3.46 ± 0.01^d^	4.79 ± 0.29^a^
Leu	1.41 ± 0.01^c^	1.73 ± 0.01^b^	1.73 ± 0.01^b^	2.77 ± 0.02^a^
Lys	2.01 ± 0.04^a^	1.91 ± 0.01^b^	1.27 ± 0.02^d^	1.64 ± 0.02^c^
Pro	1.65 ± 0.07^c^	2.02 ± 0.09^b^	2.03 ± 0.05^b^	2.87 ± 0.15^a^
TAA (mg/g)	51.35 ± 0.38^b^	48.20 ± 0.26^c^	45.41 ± 0.08^d^	63.16 ± 0.54^a^
Average Mw (kDa)	308.62	118.16	13.41	12.26

Monosaccharide composition (%)				
Mannose	6.23	4.45	5.81	8.03
Rhamnose	7.62	11.13	12.09	12.50
Galacturonic acid	24.80	16.05	19.27	21.90
Glucose	16.43	11.84	10.30	10.21
Galactose	25.72	30.81	28.27	26.06
Arabinose	19.19	25.73	24.26	21.29

Values are mean ± SD (*n* = 3). Different superscript lowercase letters within the same row indicate significant differences among fractions (*P* < 0.05). Abbreviations: TAA, total amino acids; Mw, apparent average molecular weight.

All four fractions contained more than 70% total carbohydrates (neutral sugars plus uronic acids), with uronic acids consistently exceeding neutral sugars, indicating that the preparations were dominated by acidic heteropolysaccharide systems. Although neutral sugar and uronic acid contents varied only moderately among treatments, HFTPS showed the highest values for both indices. Such differences may reflect not only altered polysaccharide release during processing, but also changes in the relative co-extraction of non-carbohydrate components (Gaur & Gänzle, 2023; Huang & Yu, 2024; Liu et al., 2025).

The polyphenol content differed markedly among the four fractions. Golden-flower fermentation alone slightly reduced the polyphenol content of LTPS, whereas pile-fermentation markedly increased it, and the highest value was observed in HFTPS. This pattern suggests that the present samples were not highly purified polysaccharides, but rather polysaccharide-enriched complexes containing varying levels of co-extracted phenolic constituents. During prolonged pile-fermentation and ageing, oxidation, polymerisation, and non-covalent association of polyphenols may increase their retention within polysaccharide-rich fractions (Guo et al., 2025; Liu et al., 2025; Xiang et al., 2023). By contrast, the shorter golden-flower stage may simultaneously promote both release and microbial consumption or transformation of phenolic compounds, resulting in a more variable net effect (Guo et al., 2025; Yu et al., 2024).

Protein contents ranged from 4.38% to 5.98%, and total amino acids ranged from 45.41 to 63.16 mg/g. These data indicate that all four fractions retained measurable nitrogen-containing components and should be regarded as polysaccharide-enriched composite systems rather than purified neutral polymers. The highest protein and total amino acid contents were observed in HFTPS, suggesting that the combined processing route enhanced the retention and/or generation of polysaccharide-associated nitrogenous constituents. Fungal growth and fermentation-related proteolysis may contribute to the release of amino acids and peptides, some of which may remain associated with polysaccharide fractions during extraction and purification (Li et al., 2023; Wang et al., 2025b).

Taken together, the four fractions shared the characteristics of acidic, uronic acid-rich tea polysaccharides, but differed substantially in the abundance of *co*-extracted polyphenols and nitrogenous components. Among them, HFTPS combined higher uronic acid, polyphenol, protein, and amino acid contents, suggesting a more complex composite structure and potentially stronger biological activity.

### 3.2 Structural characterization of tea polysaccharides

#### 3.2.1 Molecular weight distribution and monosaccharide composition

**Fig. 2:**
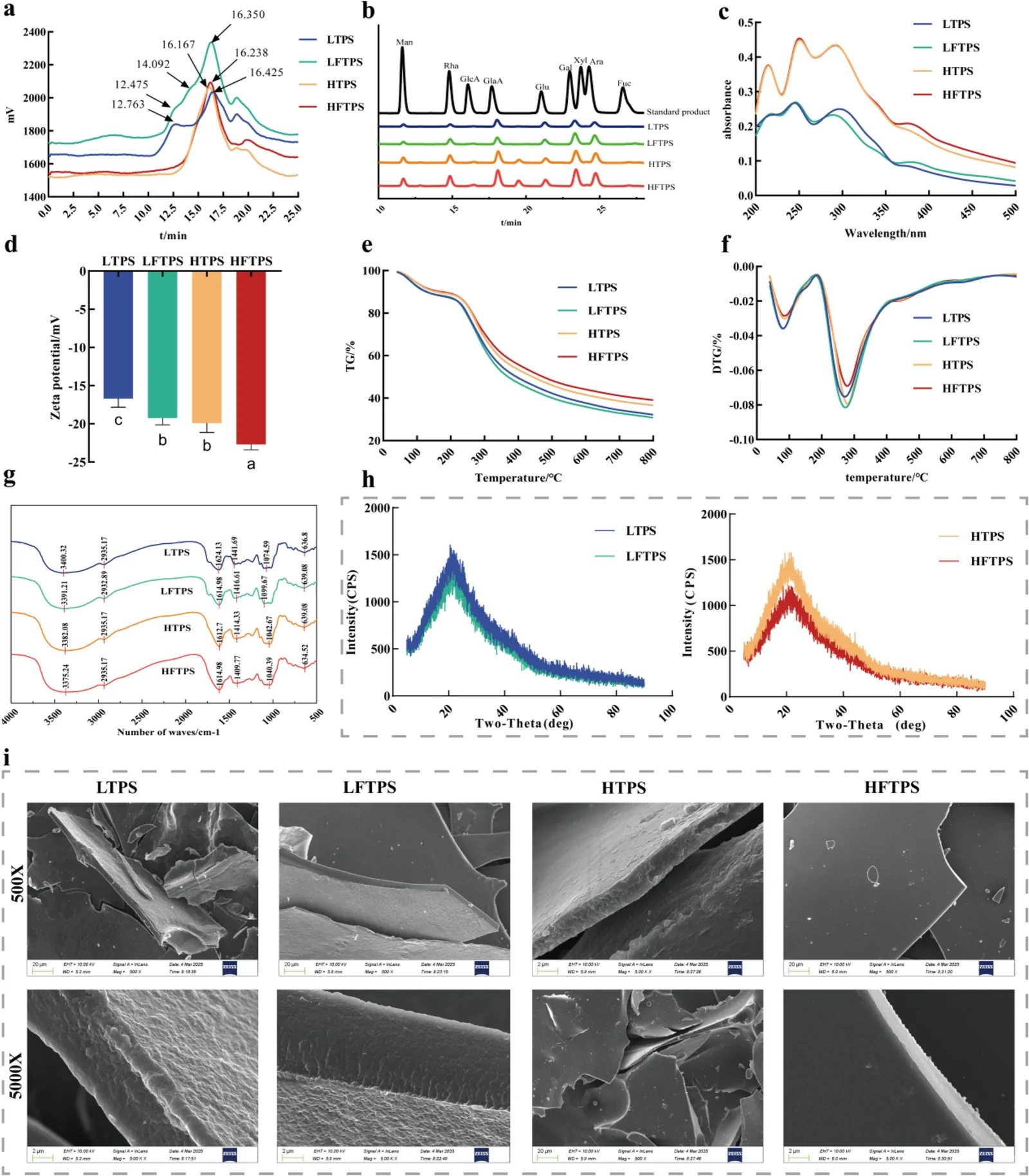
Structural characterization of tea polysaccharides from four dark teas. (a) Molecular weight distribution determined by gel permeation chromatography (GPC); (b) Monosaccharide composition analysed by PMP-HPLC; (c) UV–visible absorption spectra; (d) Zeta potential; (e) Thermogravimetric analysis (TGA); (f) Derivative thermogravimetry (DTG); (g) Fourier transform infrared (FT-IR) spectra; (h) X-ray diffraction (XRD) patterns; (i) Scanning electron microscopy (SEM) images at 500× and 5000× magnifications. LTPS, LFTPS, HTPS, and HFTPS represent polysaccharides extracted from raw dark tea (Maocha), flowered dark tea, pile-fermented dark tea, and pile-fermented and flowered dark tea, respectively.

The apparent molecular weights of the four tea polysaccharides are shown in Fig. 2a. LTPS displayed the highest apparent molecular weight (308.62 kDa), followed by LFTPS (118.16 kDa), whereas HTPS and HFTPS were concentrated in the much lower molecular-weight range (13.41 and 12.26 kDa, respectively). These results indicate that both processing steps reduced the apparent molecular size of the polysaccharide fractions, with pile-fermentation exerting the dominant effect and golden-flower fermentation further refining the low-molecular-weight profile. Fermentation-associated depolymerisation of plant polysaccharides has been widely reported and is generally attributed to microbial glycosidase activity, acid-mediated cleavage, and structural rearrangement during processing (Huang & Yu, 2024; Qi et al., 2026).

Because the present samples retained measurable amounts of polyphenols and proteins, the GPC-derived values should be interpreted as apparent molecular weights rather than absolute values. In composite systems, intermolecular association and changes in hydrodynamic volume can affect elution behaviour. Nevertheless, the overall trend is clear that pile-fermentation, especially when followed by golden-flower fermentation, shifted the polysaccharide system towards a lower molecular-weight range. Lower molecular-weight polysaccharides often show improved solubility, greater conformational flexibility, and better accessibility of reactive groups, all of which may contribute to enhanced bioactivity (Gaur & Gänzle, 2023).

As shown in Fig. 2b and Table 1, all four fractions shared the same monosaccharide species, namely mannose, rhamnose, galacturonic acid, glucose, galactose, and arabinose, indicating that processing did not fundamentally alter the monosaccharide repertoire of the tea polysaccharide system. However, the molar ratios changed considerably. Compared with LTPS, both LFTPS and HTPS showed lower proportions of mannose, galacturonic acid, and glucose, together with higher proportions of rhamnose, galactose, and arabinose. Relative to HTPS, HFTPS showed increased mannose, rhamnose, and galacturonic acid, but decreased glucose, galactose, and arabinose.

These results suggest that fermentation mainly reshaped the relative abundance of specific structural domains rather than replacing the overall carbohydrate framework. The relatively high galacturonic acid content confirms the acidic nature of the fractions, whereas the co-occurrence of rhamnose, galactose, and arabinose implies the presence of branched side-chain regions. Acidic groups and monosaccharide profile are widely recognised as important determinants of polysaccharide charge properties, solution behaviour, and biological function (Tan et al., 2024). In the present study, the higher mannose and galacturonic acid proportions observed in HFTPS may partly contribute to its stronger functional performance, although these effects are likely to arise from the combined influence of composition, molecular size, and co-retained components rather than any single structural variable.

#### 3.2.2 Spectroscopic, colloidal, thermal and morphological characteristics

The UV–Vis spectra of the four fractions are shown in Fig. 2c. All samples displayed noticeable absorption in the 200–260 nm region, together with a weak band near 280 nm, indicating the presence of *co*-extracted non-polysaccharide chromophores, such as proteinaceous and phenolic constituents (Wei et al., 2023; Yang et al., 2025). In addition, absorption around 380 nm was more pronounced in the flowering-related fractions, especially HFTPS, suggesting a higher retention of pigment-related oxidised polyphenolic components, such as theabrownin-like substances, within the polysaccharide-enriched matrix (Chen et al., 2024b; Liu et al., 2025).

The zeta potentials ranged from approximately −15 to −25 mV **(**Fig. 2d**)**, confirming that all samples carried net negative charges in aqueous dispersion, consistent with their high uronic acid content. Pile-fermentation and golden-flower fermentation both increased the absolute value of the negative zeta potential, and HFTPS exhibited the highest absolute potential. A larger absolute zeta potential generally indicates stronger electrostatic repulsion between particles and better colloidal stability (Xia et al., 2025; Zhou et al., 2025). This feature may favour more homogeneous dispersion of HFTPS in aqueous systems and contribute to its stronger *in vitro* performance.

The TGA and DTG profiles are shown in Fig. 2e–f. All fractions displayed the typical three-stage thermal decomposition pattern of carbohydrate-rich biopolymers, including water loss at low temperature, primary polysaccharide degradation at intermediate temperature, and gradual decomposition of carbonaceous residues at higher temperature (Guo et al., 2025; Wang et al., 2025a). Compared with LTPS, the main degradation events of LFTPS, HTPS, and HFTPS shifted towards higher temperatures, indicating improved thermal stability after processing. Among them, HFTPS showed the lowest mass-loss rate in the main decomposition region, suggesting a more thermally stable matrix.

The FT-IR spectra **(**Fig. 2g**)** of all four fractions were broadly similar, indicating that the main polysaccharide backbone was preserved after processing. The broad band at approximately 3377–3394 cm^−1^ corresponded to O—H stretching, the bands at approximately 1614–1625 cm^−1^ and 1414–1442 cm^−1^ were attributable to carboxylate-related vibrations, and the region around 1040–1101 cm^−1^ reflected C—O and glycosidic-linkage vibrations. These are typical features of acidic polysaccharides (Yu et al., 2024; Zhou et al., 2025). The subtle shifts in band position among the four samples suggest that fermentation altered hydrogen-bonding patterns, ionisation state, or the microenvironment of functional groups, rather than the overall polysaccharide backbone.

The XRD patterns **(**Fig. 2h**)** showed broad diffraction halos centred near 21° (2θ), without sharp crystalline peaks, indicating that all fractions were predominantly amorphous. The weaker diffraction intensity of LFTPS and HFTPS relative to LTPS and HTPS suggests that golden-flower fermentation may reduce local structural ordering and increase amorphous character. Similar decreases in crystallinity or local packing order have been reported in fermentation-modified polysaccharide systems (Lee et al., 2024; Wei et al., 2023).

SEM images **(**Fig. 2i**)** further supported the processing-induced structural differences. At low magnification, all samples appeared as lamellar or flake-like aggregates, but HTPS and HFTPS showed smoother and more compact surfaces than LTPS and LFTPS. At higher magnification, HFTPS exhibited the densest and least porous morphology. In dried polysaccharide systems, such differences often reflect altered molecular packing and intermolecular association during dehydration (Falsafi et al., 2025; Zhang et al., 2024b). Although SEM does not represent the native solution-state conformation, the denser morphology of HFTPS is consistent with its stronger inter-component association and distinct colloidal behaviour.

Overall, the structural analyses indicate that pile-fermentation and golden-flower fermentation did not change the basic acidic heteropolysaccharide nature of the tea polysaccharide system, but markedly reshaped its molecular-size distribution, monosaccharide ratios, surface charge, thermal stability, and dry-state morphology. HFTPS combined the lowest apparent molecular weight, the highest absolute zeta potential, stronger thermal stability, and a denser microstructure, supporting its designation as the fraction with the most distinctive integrated structural characteristics among the four samples.

### 3.3 *In vitro* bioactivities of polysaccharides

#### 3.3.1 Antioxidant activity

**Fig. 3:**
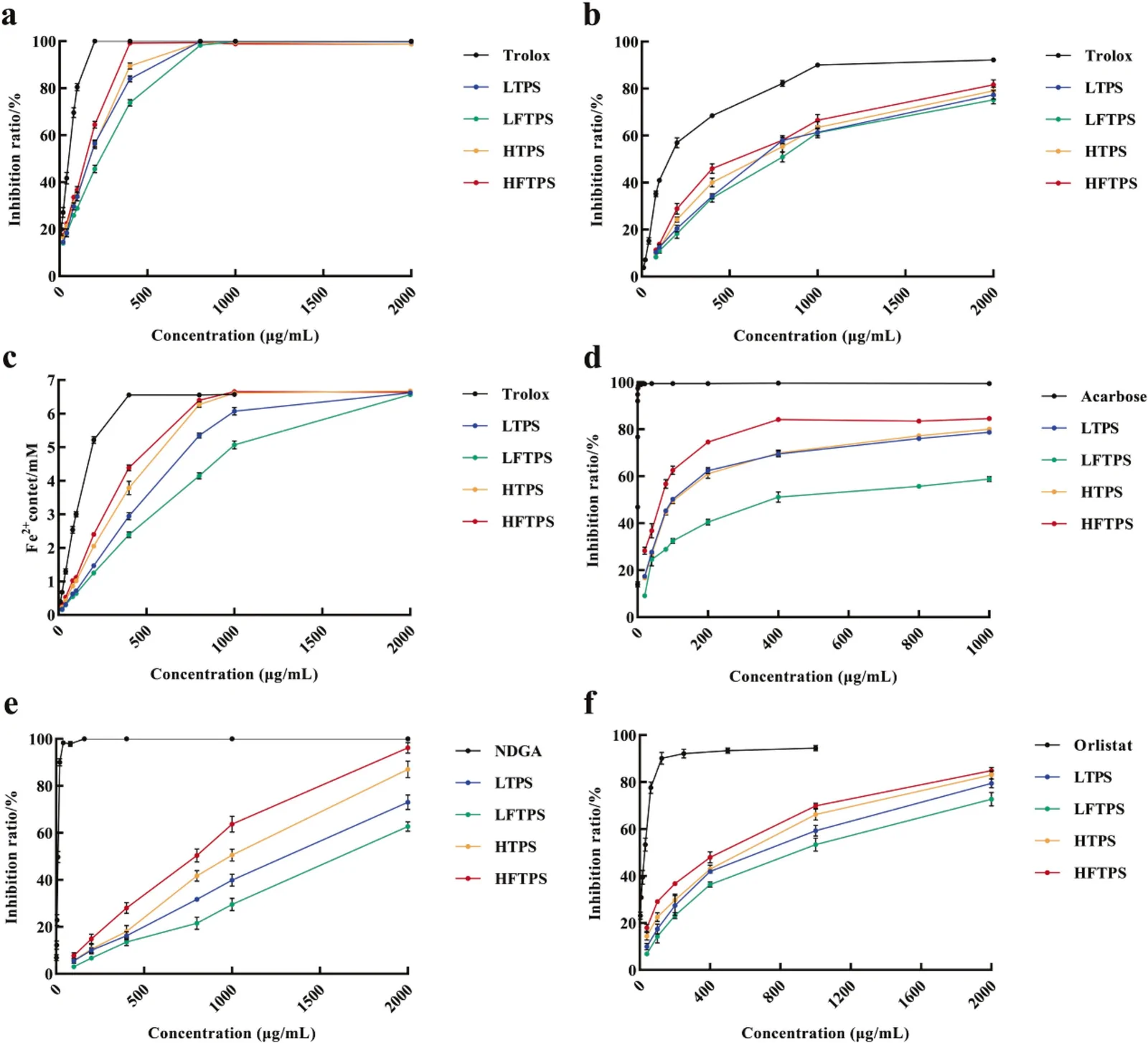
*In vitro* antioxidant and enzyme inhibitory activities of tea polysaccharides from four dark teas. (a) ABTS radical scavenging activity; (b) DPPH radical scavenging activity; (c) Ferric reducing antioxidant power (FRAP); (d) α-glucosidase inhibitory activity; (e) Lipoxygenase inhibitory activity; (f) Cholesterol esterase inhibitory activity. Trolox was used as a positive control for antioxidant assays (a–c), acarbose for α-glucosidase inhibition (d), nordihydroguaiaretic acid (NDGA) for lipoxygenase inhibition (e), and orlistat for cholesterol esterase inhibition (f). Data are expressed as mean ± SD (*n* = 3).

As shown in Fig. 3a–c, all four tea polysaccharide fractions exhibited concentration-dependent antioxidant activity in the ABTS, DPPH, and FRAP assays. Although their activities were lower than those of Trolox, clear differences were observed among the fractions. In both radical-scavenging assays, HFTPS showed the strongest activity, followed by HTPS, LTPS, and LFTPS. The same trend was observed for ferric reducing power, indicating that the processing-dependent differences were reproducible across different antioxidant assays.

The lower activity of LFTPS relative to LTPS suggests that golden-flower fermentation alone did not necessarily enhance antioxidant capacity under the low pile-fermentation background. By contrast, pile-fermentation substantially improved antioxidant performance, and the sequential processing route was associated with a further increase in activity. This pattern is consistent with the structural characteristics described above, especially the lower apparent molecular weight, higher polyphenol content, and more negative surface charge of HFTPS. In polysaccharide-rich composite systems, antioxidant capacity is often governed by multiple coexisting factors, including molecular size, uronic acid content, and associated phenolic constituents (Gaur & Gänzle, 2023; Xiang et al., 2023). Therefore, the superior antioxidant activity of HFTPS is more plausibly explained by overall process-driven structural remodelling than by any single component.

Oxidative stress is closely linked to lipid accumulation and metabolic dysfunction, because excessive reactive oxygen species can trigger lipid peroxidation and damage cell membranes and metabolic enzymes (Tan et al., 2024). The stronger antioxidant performance of HTPS and especially HFTPS therefore provides a plausible functional basis for their subsequent effects on lipid-related phenotypes in *C. elegans*.

#### 3.3.2 Inhibitory activities against key metabolic enzymes

The inhibitory activities against α-glucosidase, lipoxygenase, and cholesterol esterase are shown in Fig. 3d–f. All four fractions inhibited these enzymes in a concentration-dependent manner, but the strength of inhibition differed markedly among samples. For α-glucosidase, HFTPS showed the lowest IC₅₀ value and therefore the strongest inhibitory activity, whereas LFTPS showed the weakest effect. HTPS and LTPS displayed intermediate inhibition. A similar ranking was observed for lipoxygenase and cholesterol esterase inhibition, with HFTPS consistently performing best. The calculated IC₅₀ values for α-glucosidase inhibition were 99.17, 379.30, 104.79, and 66.49 μg/mL for LTPS, LFTPS, HTPS, and HFTPS, respectively. The corresponding IC₅₀ values for lipoxygenase inhibition were 1306.53, 1618.09, 988.68, and 794.03 μg/mL, whereas those for cholesterol esterase inhibition were 678.72, 880.42, 582.54, and 455.63 μg/mL, respectively. For comparison, the IC₅₀ values of the corresponding positive controls were 0.0553 μg/mL for acarbose, 10.08 μg/mL for NDGA, and 27.45 μg/mL for orlistat. Among the four polysaccharide fractions, HFTPS consistently exhibited the lowest IC₅₀ value for all three enzymes, indicating the strongest overall inhibitory potency.

The relevance of these assays lies in their connection to metabolic regulation. α-Glucosidase inhibition can reduce glucose release from dietary carbohydrates and may attenuate postprandial glycaemic burden (Li et al., 2022). Lipoxygenase participates in the production of lipid-derived inflammatory mediators and has been implicated in inflammation-associated lipid dysregulation (Haeggström & Funk, 2011). Cholesterol esterase contributes to intestinal processing of dietary lipids and cholesterol esters, and its inhibition has been proposed as one route to limit lipid absorption (Yu et al., 2025). The fact that HFTPS showed the strongest inhibition across all three targets suggests that this fraction possessed broader metabolic regulatory potential than the other samples.

As with the antioxidant assays, these differences are unlikely to arise from a single structural feature. Lower apparent molecular weight may improve access to enzyme surfaces, while higher uronic acid content, stronger negative charge, and greater retention of polyphenol- and protein-associated components may favour multiple non-covalent interactions with enzymes (Chen et al., 2025b; Liu et al., 2025). Although direct binding studies would be needed to confirm these interactions, the present data indicate that the processing route influenced the *in vitro* functional profile of the tea polysaccharide fractions.

### 3.4 *In vivo* phenotypes, lipid accumulation, lifespan and stress resistance

**Fig. 4:**
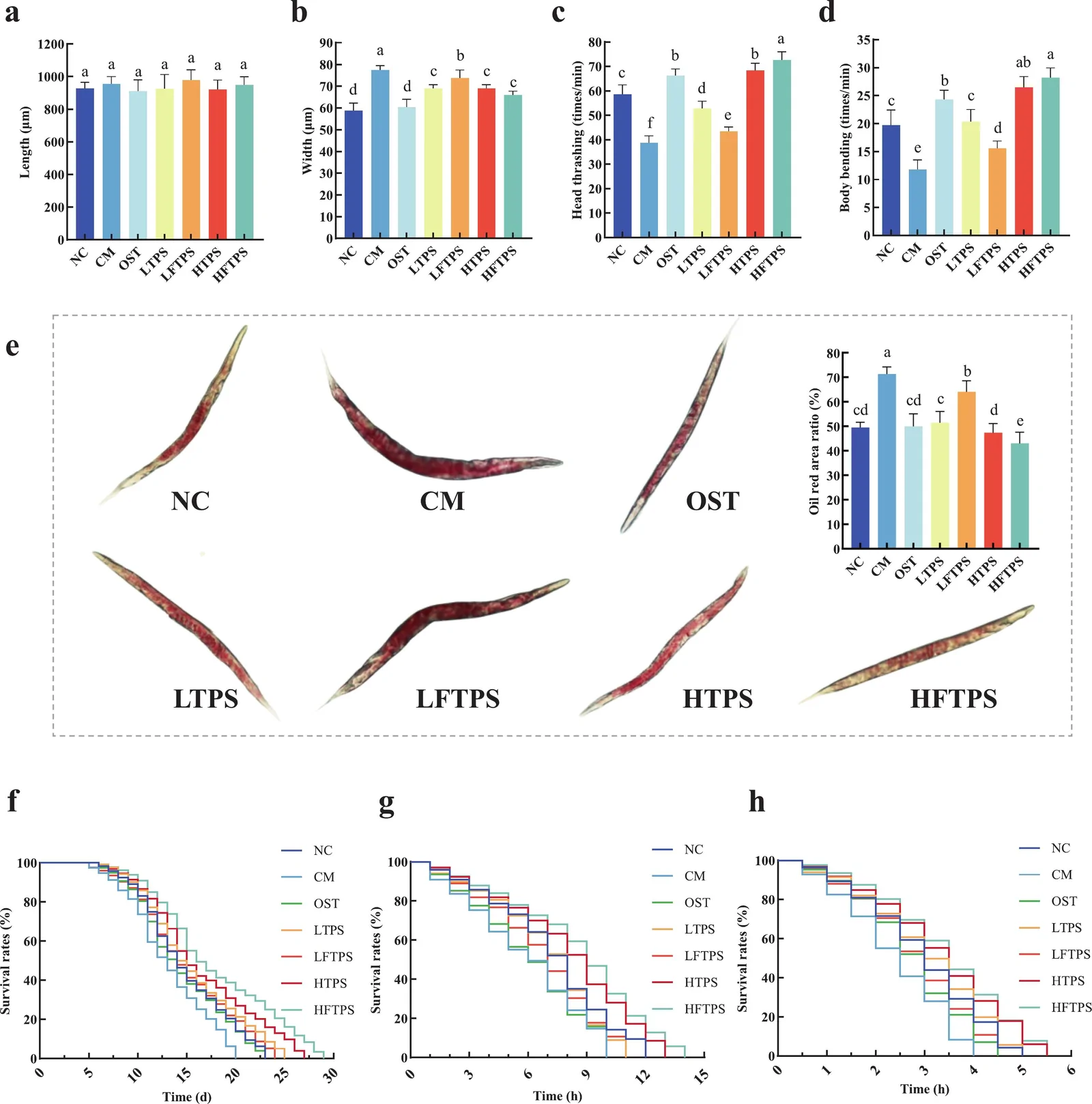
Effects of tea polysaccharides on *in vivo* phenotypes, lipid accumulation, lifespan, and stress resistance in high-glucose-induced *Caenorhabditis elegans*. (a) Body length; (b) Body width; (c) Head thrashing frequency; (d) Body bending frequency; (e) Representative Oil Red O staining images and quantification of lipid accumulation; (f) Lifespan curves under normal conditions; (g) Survival curves under heat stress (35 °C); and (h) Survival curves under oxidative stress induced by H₂O₂. Data are expressed as mean ± SD where applicable. Different letters indicate significant differences among groups (*P* < 0.05). For panels a–e, groups that do not share a lowercase letter differ significantly at *P* < 0.05; asterisks are not used. Survival differences in panels f–h were analysed using the log-rank (Mantel–Cox) test. (For interpretation of the references to colour in this figure legend, the reader is referred to the web version of this article.)

A high-glucose *C. elegans* model was used to assess whether the *in vitro* activities of the tea polysaccharides translated into improved *in vivo* phenotypes. High-glucose exposure is commonly used to induce obesity-like phenotypes and metabolic stress in *C. elegans* (Berk et al., 2024). Compared with the normal control, the model control showed little change in body length but a clear increase in body width **(**Fig. 4a–b**)**, consistent with enhanced lipid burden. All four polysaccharide treatments reduced body width relative to the model group, and the strongest effect was observed in the HFTPS-treated group.

Locomotor performance was also impaired by high-glucose exposure. Both head-thrashing frequency and body-bending frequency were significantly decreased in the model group **(**Fig. 4c–d**)**, indicating reduced health status and neuromuscular performance. Treatment with tea polysaccharides improved both locomotor indices to varying extents, again with HFTPS showing the most pronounced recovery. In *C. elegans*, locomotion is widely used as an integrative readout of physiological status and healthspan (An et al., 2023; Berk et al., 2024). The improvement in locomotor performance observed here therefore supports a broader protective effect of the tea polysaccharides against glucose-induced functional decline.

Oil Red O staining confirmed that high-glucose treatment promoted marked lipid accumulation **(**Fig. 4e**)**. Compared with the model group, all four polysaccharide fractions significantly reduced the Oil Red O-positive area, indicating suppression of neutral lipid deposition. This result is consistent with the morphological changes in body width and suggests that the observed phenotypic improvements were closely linked to reduced lipid burden. Among the fractions, HFTPS produced the greatest decrease in lipid staining, whereas LFTPS showed the weakest response.

High-glucose treatment also shortened lifespan and reduced resistance to heat and oxidative stress (Fig. 4f–h). Relative to the normal control, the survival curves of the model group were shifted to the left under normal, heat-stress, and oxidative-stress conditions, demonstrating that glucose-induced metabolic burden impaired overall viability and stress tolerance. All four tea polysaccharides improved survival to different extents, with HFTPS and HTPS showing the strongest benefits. The consistent improvement in lifespan, heat-stress resistance, and oxidative-stress resistance suggests that the tea polysaccharides did not simply reduce lipid storage, but also enhanced physiological robustness under stress conditions.

Taken together, the *in vivo* data demonstrate that tea polysaccharides alleviated multiple consequences of high-glucose exposure, including increased body width, reduced locomotion, lipid accumulation, shortened lifespan, and impaired stress resistance. The superiority of HFTPS across these endpoints agrees well with its integrated structural and *in vitro* functional advantages, indicating that the fraction obtained after pile-fermentation followed by golden-flower fermentation showed the strongest overall *in vivo* performance.

### 3.5 Biochemical parameters and lipid metabolism-related gene expression

#### 3.5.1 Oxidative stress- and lipid-related biochemical indices

**Fig. 5:**
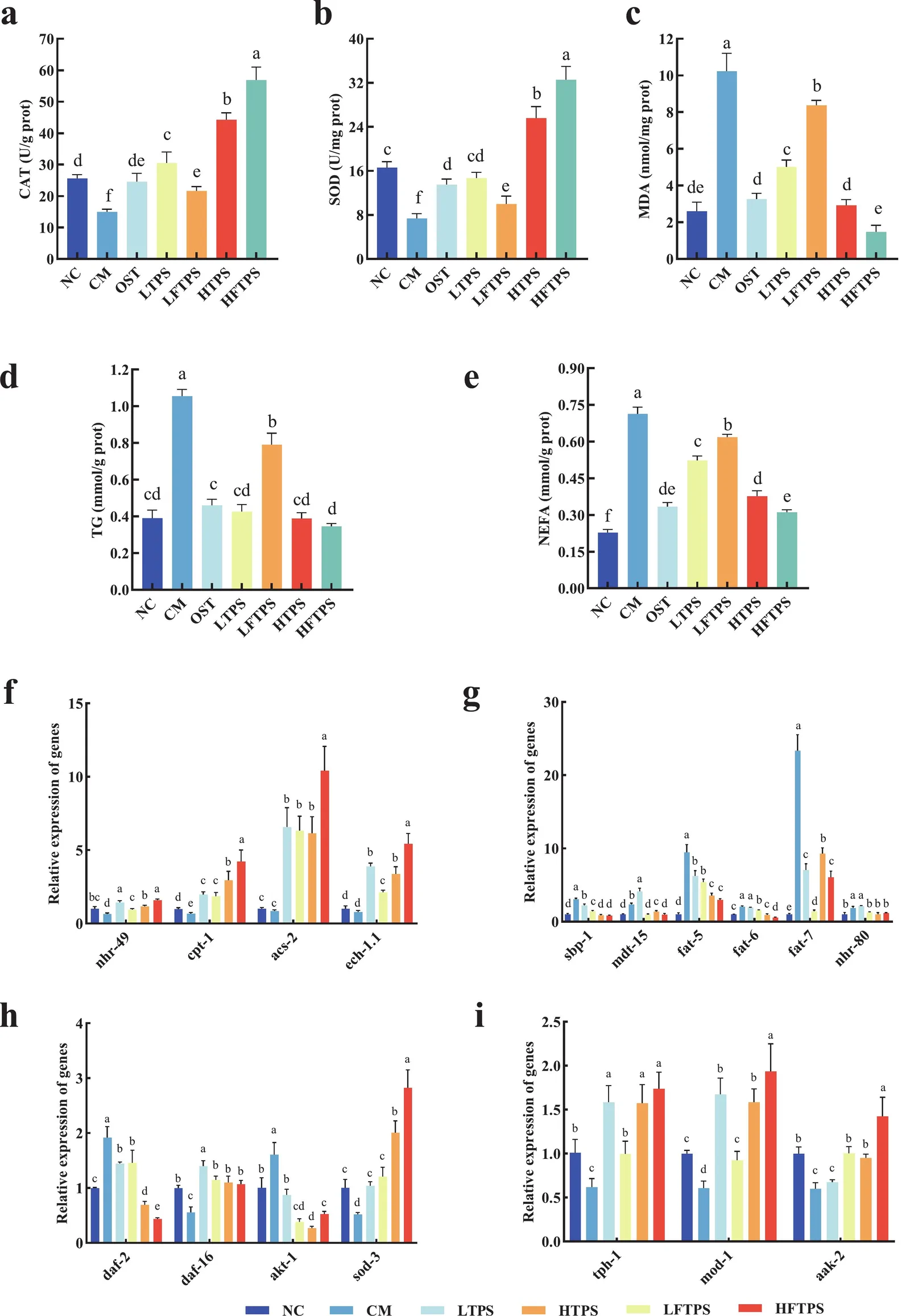
Effects of tea polysaccharides on biochemical parameters and lipid metabolism-related gene expression in *Caenorhabditis elegans*. (a) Superoxide dismutase (SOD) activity; (b) Catalase (CAT) activity; (c) Malondialdehyde (MDA) content; (d) Triglyceride (TG) content; (e) Non-esterified fatty acid (NEFA) content; (f) Genes involved in fatty acid β-oxidation and nuclear receptor signalling (*e.g.*, *nhr-49*, *cpt-1*, *ech-1.1*, and *acs-2*); (g) Genes related to lipid synthesis (*e.g.*, *sbp-1*, *fat-5*, *fat-6*, and *fat-7*); (h) Genes associated with the insulin/IGF-1 signalling pathway (*e.g.*, *daf-2*, *daf-16*, and *akt-1*); and (i) Genes involved in neurotransmission and energy metabolism (*e.g.*, *tph-1*, mod*-1*, and *aak-2*). Synchronised worms were treated for 3 days under high-glucose conditions before biochemical and transcriptional analyses. Gene expression levels were normalized to the internal control gene *act-1* and calculated using the 2^−ΔΔCt method. Data are presented as mean ± SD (*n* = 3). Different letters indicate significant differences among groups (*P* < 0.05). Groups that do not share a lowercase letter differ significantly at *P* < 0.05; asterisks are not used.

To further validate the *in vivo* effects of tea polysaccharides, oxidative stress-related enzymes and lipid-associated biochemical indices were measured in *C. elegans* (Fig. 5a–e). Compared with the normal control, the model group showed significantly lower SOD and CAT activities, together with markedly higher TG, NEFA, and MDA levels. These changes indicate that high-glucose exposure impaired endogenous antioxidant defence, increased lipid burden, and enhanced lipid peroxidation.

All four tea polysaccharide fractions improved these biochemical indices to varying degrees. SOD and CAT activities increased significantly after polysaccharide treatment, whereas TG, NEFA, and MDA were reduced relative to the model group. The most pronounced overall improvement was again observed in the HFTPS group. These data are consistent with the phenotypic observations in Fig. 4 and indicate that the protective effect of tea polysaccharides involved both attenuation of oxidative stress and alleviation of lipid metabolic imbalance.

The decrease in MDA is particularly relevant because MDA is a widely used marker of lipid peroxidation and oxidative membrane damage (Berk et al., 2024; Valgimigli, 2023). In parallel, the recovery of SOD and CAT activity suggests that the tea polysaccharides helped restore endogenous antioxidant defence capacity (Xiao et al., 2023; Yan et al., 2018). The coordinated reduction in TG and NEFA, together with decreased Oil Red O staining, further confirms that the fractions, especially HFTPS, effectively mitigated glucose-induced lipid accumulation.

Oxidative stress, inflammation, insulin resistance, and lipid accumulation can reinforce one another in a feed-forward cycle. Excess reactive oxygen species promote lipid peroxidation and activate stress-sensitive inflammatory signalling, while inflammatory mediators and mitochondrial dysfunction can impair insulin/IGF and AMPK control, suppress fatty acid oxidation, and favour lipogenesis. Conversely, lipid overload can further increase mitochondrial reactive oxygen species and inflammatory mediator production. Evidence from complementary models links hepatic lipid accumulation to disruption of the AMPK/ACC axis (Ling et al., 2023), connects mitochondrial dysfunction with altered lipid-droplet homeostasis (Chen et al., 2024a), and shows that oxidative and inflammatory pathways involving NF-κB/MAPK or JAK2/STAT3 can aggravate tissue injury (Xu et al., 2023; Zhang et al., 2024a). Gut-microbiota perturbation has likewise been associated with abnormal lipid metabolism (Zhang et al., 2022). In the present study, the coordinated recovery of SOD and CAT, reduction of MDA, TG, NEFA, and Oil Red O staining, and normalization of oxidation- and lipogenesis-related transcripts are consistent with attenuation of this cycle. However, inflammatory mediators were not measured directly; therefore, an anti-inflammatory contribution remains a testable mechanistic hypothesis rather than a demonstrated pathway.

#### 3.5.2 Regulation of lipid metabolism-related gene expression

To explore whether the phenotypic and biochemical improvements were accompanied by transcriptional responses in lipid metabolism-related pathways, RT–qPCR was used to quantify representative genes involved in fatty acid oxidation, lipogenesis, insulin/IGF-related signalling, and serotonin/AMPK-related regulation (Fig. 5f–i).

The genes *nhr-49*, *cpt-1*, *ech-1.1*, and *acs-2* are associated with fatty acid β-oxidation and nuclear receptor signalling. In the high-glucose model, these genes were generally downregulated relative to the normal control, suggesting suppression of lipid catabolism. Tea polysaccharide treatment reversed this trend to different extents, and HFTPS showed the strongest overall upregulation. In *C. elegans*, *nhr-49* is a central regulator of fatty acid utilisation and has often been regarded as functionally analogous to mammalian PPARα (Jin et al., 2025). The increased expression of *nhr-49* and its downstream oxidation-related genes is therefore consistent with enhanced fatty acid breakdown and reduced lipid storage.

By contrast, the lipogenesis-related genes *sbp-1*, *fat-5*, *fat-6*, and *fat-7* were generally elevated in the high-glucose model and were suppressed after polysaccharide treatment, particularly in the HTPS and HFTPS groups. *Sbp-1* is the *C. elegans* homologue of SREBP and is a key transcriptional regulator of lipid synthesis, whereas *fat-5*, *fat-6*, and *fat-7* encode stearoyl-CoA desaturase-like enzymes that contribute to fatty acid desaturation and lipid accumulation (An et al., 2023; Wan et al., 2022). The downregulation of these genes after polysaccharide treatment therefore agrees well with the observed decreases in TG, NEFA, and Oil Red O staining.

The insulin/IGF-related genes *daf-2*, *daf-16*, and *akt-1* also showed clear responses to glucose stress and polysaccharide treatment. Compared with the normal control, the model group exhibited a transcriptional pattern consistent with disturbed insulin-like signalling, whereas the polysaccharide-treated groups, especially HFTPS, shifted this pattern towards a more favourable state. The *daf-2*/*daf-16*/*akt-1* axis is a core regulatory module in *C. elegans* controlling metabolism, stress resistance, and longevity (Goyache et al., 2024; Yu et al., 2020). The expression changes observed here are therefore consistent with the improvements in lifespan, oxidative-stress tolerance, and biochemical antioxidant status.

In addition, the serotonin/AMPK-related genes *tph-1*, mod*-1*, and *aak-2* were downregulated in the model group and recovered after polysaccharide treatment, again most clearly in the HFTPS group. *Tph-1* is involved in serotonin biosynthesis, mod*-1* encodes a serotonin-gated ion channel, and *aak-2* is the catalytic subunit of AMPK in *C. elegans* (An et al., 2023). Together, these genes are linked to neurometabolic regulation, energy sensing, and stress adaptation. Their upregulation after treatment suggests that tea polysaccharides may improve lipid homeostasis not only through direct metabolic pathways, but also through broader neuroendocrine and energy-sensing regulation.

Overall, the gene expression data show a coherent transcriptional shift induced by the tea polysaccharides: promotion of β-oxidation-related genes, suppression of lipogenesis-related genes, and favourable modulation of insulin/IGF- and AMPK-related responses. The most pronounced overall transcriptional response pattern was again observed for HFTPS, which agrees with its superior structural profile, *in vitro* bioactivities, and *in vivo* protective effects.

### 3.6 Correlation analysis

**Fig. 6:**
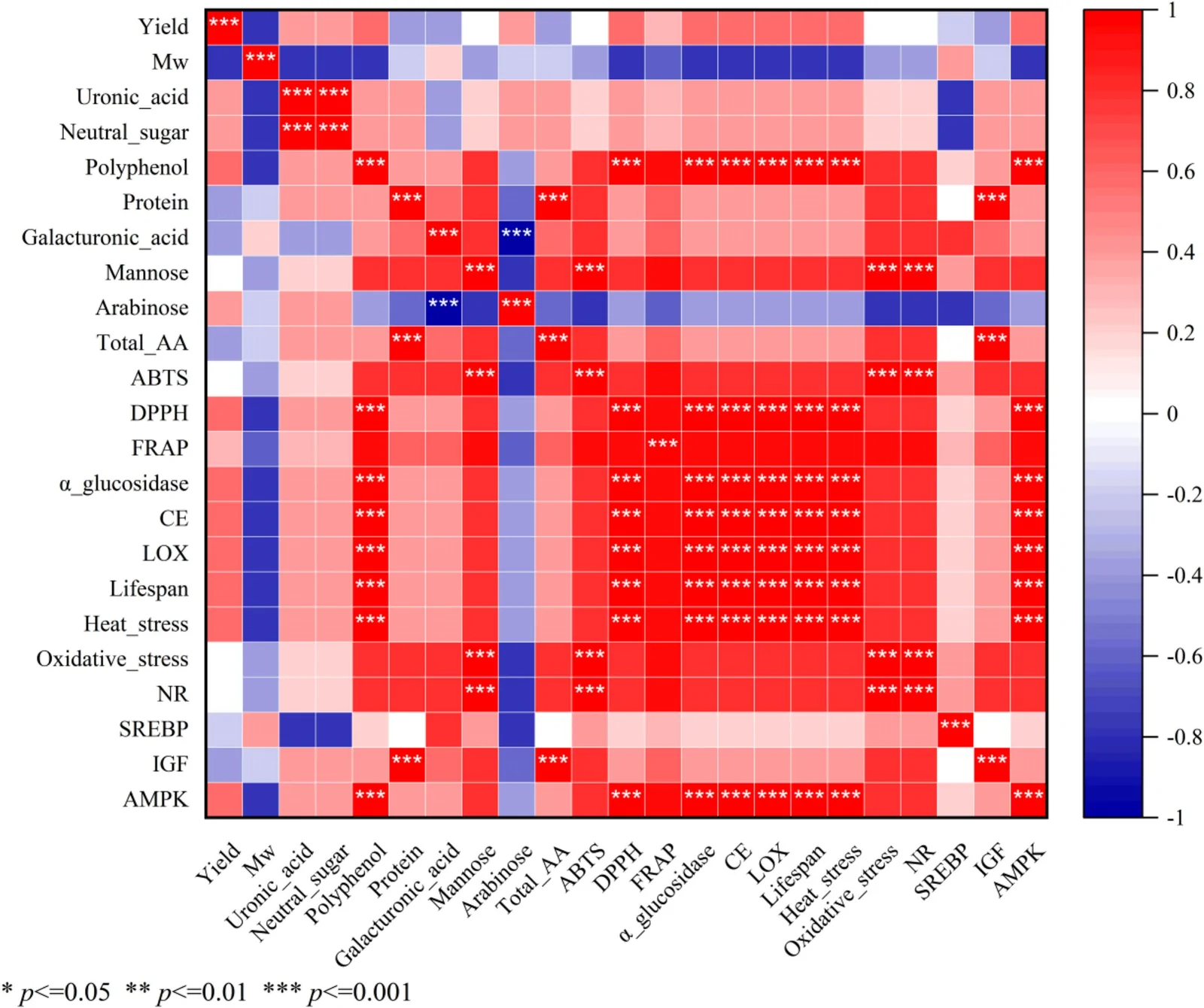
Spearman correlation heatmap of physicochemical properties, *in vitro* bioactivities, *in vivo* phenotypes, and pathway-level gene response indices of four tea polysaccharides. Colour intensity represents the Spearman correlation coefficient (*r*), ranging from −1 to 1, with red and blue indicating positive and negative correlations, respectively. Asterisks indicate significant correlations (*P < 0.05, **P < 0.01, ***P < 0.001). CE, cholesterol esterase inhibitory activity; LOX, lipoxygenase inhibitory activity; NR, nuclear receptor pathway index; SREBP, sterol regulatory element-binding protein pathway index; IGF, insulin/IGF-related pathway index; AMPK, serotonin–AMPK-related pathway index. (For interpretation of the references to colour in this figure legend, the reader is referred to the web version of this article.)

To further explore potential links among structural characteristics, *in vitro* bioactivities, and *in vivo* responses, a Spearman correlation heatmap was generated using representative physicochemical variables, functional indices, phenotypic outcomes, and pathway-level gene response indices (Fig. 6). Because only four polysaccharide fractions were compared, this analysis was used primarily to visualise co-varying trends rather than to establish robust structure–function relationships or strict causality. The Pearson correlation matrix showed a broadly similar overall pattern and was therefore provided as supplementary validation (**Fig. S1**).

The heatmap revealed a clear modular distribution. Polyphenol content, mannose proportion, total amino acids, and acidic components were generally positively associated with antioxidant activity, enzyme inhibitory activity, lifespan, stress resistance, and the NR/AMPK pathway indices, whereas apparent molecular weight generally showed the opposite trend. These patterns suggest that lower apparent molecular weight and a higher abundance of associated acidic and non-carbohydrate components may be related to stronger overall bioactivity.

Among the structural variables, polyphenol content showed strong positive associations with ABTS, DPPH, FRAP, and inhibition of α-glucosidase, cholesterol esterase, and lipoxygenase. This is consistent with the widely reported contribution of polysaccharide-associated polyphenols to antioxidant and metabolic regulatory functions (Huang & Yu, 2024; Tan et al., 2024). In contrast, apparent molecular weight was negatively associated with most bioactivity indices, supporting the view that lower-molecular-weight fractions tend to display better accessibility and stronger functional expression (Huang & Yu, 2024; Lee et al., 2024).

Mannose and galacturonic acid also showed generally positive associations with several functional and phenotypic indices, whereas arabinose tended to show the opposite pattern (Qi et al., 2026; Tan et al., 2024). In addition, protein and total amino acids were positively associated with multiple bioactivity-related variables, further supporting the interpretation that the present samples behaved as polysaccharide–protein–polyphenol complexes rather than purified polysaccharides (Huang & Yu, 2024).

At the phenotype level, antioxidant and enzyme inhibitory activities tended to be positively associated with lifespan, heat-stress resistance, and oxidative-stress resistance. Lifespan and stress-resistance indices also showed generally positive associations with the NR and AMPK pathway indices and negative associations with the SREBP pathway index. These trends are consistent with the overall observation that the more active fractions, particularly HFTPS, were also associated with a more favourable lipid-related and stress-response phenotype in *C. elegans*.

Taken together, the correlation analysis provides exploratory support for a link between processing-associated structural variation and functional performance. However, these associations should be interpreted cautiously because of the limited number of fractions analysed. The results are therefore better viewed as trend-level evidence that helps contextualise the superior overall activity of HFTPS, rather than as definitive proof of structure–function causality.

### 3.7 Proposed structure–function interpretation

**Fig. 7:**
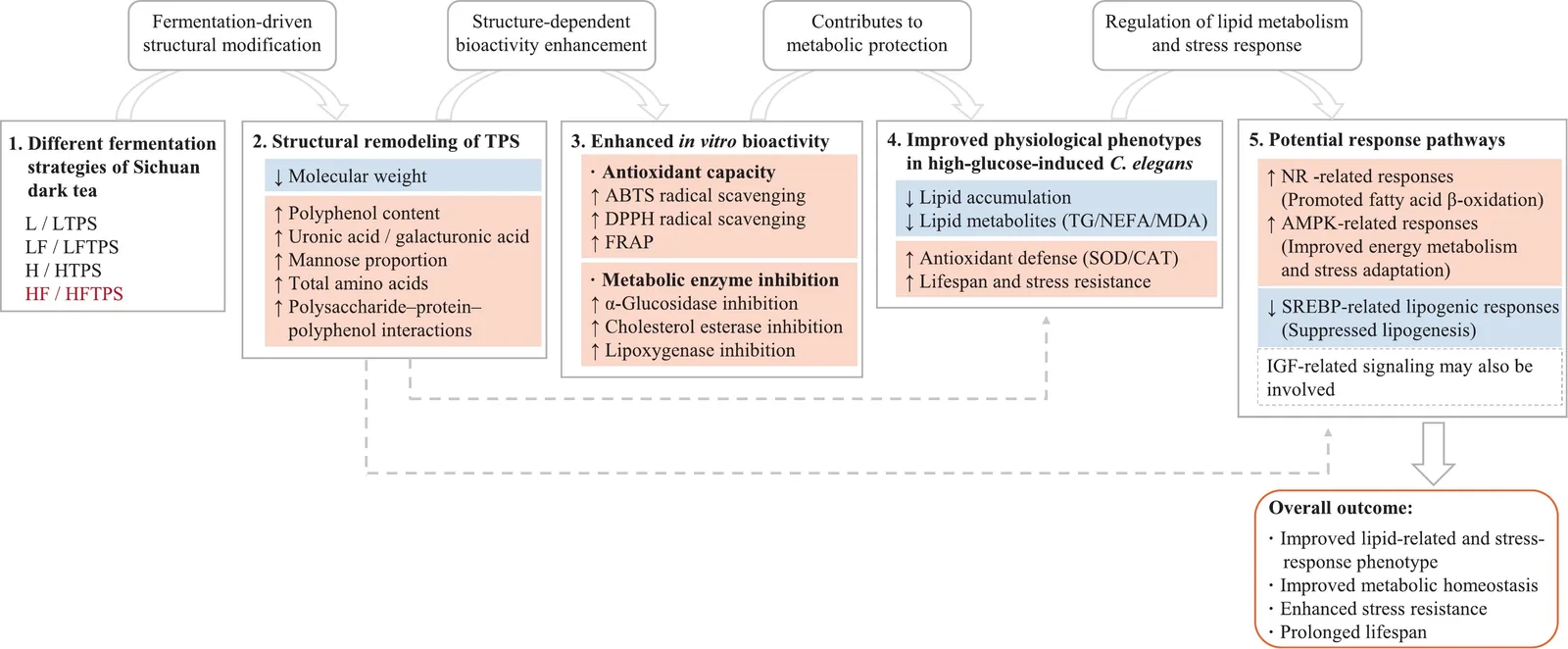
Proposed structure–function interpretation of fermented dark tea polysaccharides in relation to lipid regulation and stress resistance in *Caenorhabditis elegans*. Different fermentation strategies, particularly the sequential pile-fermentation and golden-flower fermentation process, were associated with marked structural variation in tea polysaccharides, including lower apparent molecular weight, higher polyphenol and acidic component contents, elevated mannose and total amino acid levels, and enhanced polysaccharide–protein–polyphenol interactions. These structural characteristics were associated with stronger antioxidant capacity and metabolic enzyme inhibitory activities, which in turn corresponded to reduced lipid accumulation, improved antioxidant defence, enhanced stress resistance, and prolonged lifespan in high-glucose-induced *C. elegans*. At the pathway level, the beneficial effects were associated with transcriptional response patterns consistent with enhanced NR- and AMPK-related regulation and reduced SREBP-related lipogenic responses, while IGF-related signalling may also be involved. Solid arrows indicate experimentally supported relationships, whereas dashed arrows indicate inferred associations based on exploratory correlation analysis.

Based on the integrated results of structural analysis, *in vitro* assays, *C. elegans* phenotyping, biochemical evaluation, transcriptional profiling, and exploratory correlation analysis, a proposed structure–function interpretation is summarised in Fig. 7. The data suggest that pile-fermentation and golden-flower fermentation, especially when applied sequentially, were associated with marked changes in apparent molecular-weight distribution, acidic characteristics, associated polyphenol and protein contents, surface charge, and supramolecular organisation of the tea polysaccharide-enriched fractions.

These processing-associated structural differences were accompanied by clear differences in functional performance. Fractions with lower apparent molecular weight and higher levels of associated acidic and non-carbohydrate components, especially HFTPS, showed stronger antioxidant capacity and stronger inhibition of key metabolic enzymes *in vitro*. In the high-glucose *C. elegans* model, these differences were further reflected in reduced lipid accumulation, improved locomotor performance, prolonged lifespan, and enhanced heat- and oxidative-stress resistance. At the biochemical level, the same fraction was associated with reduced TG, NEFA, and MDA levels and with higher SOD and CAT activities.

At the transcriptional level, HFTPS was associated with response patterns consistent with enhanced fatty acid β-oxidation, reduced lipogenesis, and modulation of insulin/IGF- and AMPK-related signalling. When considered together, these results suggest that the superior activity of HFTPS is more likely to arise from integrated processing-associated structural and compositional features than from any single isolated variable.

Taken together, HFTPS exhibited the most favourable integrated structural and functional profile among the four fractions, as supported by the convergence of multiple independent endpoints rather than superiority in every individual variable. HFTPS combined the lowest apparent molecular weight (12.26 kDa), the highest absolute zeta potential, and the highest uronic acid, polyphenol, protein, total amino acid, and mannose levels with the lowest IC₅₀ values for all three metabolic enzymes. It also showed the strongest overall reductions in lipid staining, TG, NEFA, and MDA, together with the greatest recovery of SOD and CAT activities and the most favourable combined responses in locomotion, lifespan, stress resistance, and lipid metabolism-related gene expression. This cross-level consistency supports HFTPS as the strongest overall-performing fraction in the present study.

It should be emphasised that Fig. 7 is intended as a conceptual summary of the observed cross-level relationships rather than as direct proof of causal signalling interactions. The pathway-level interpretation is based mainly on transcriptional responses and consistency among structural, biochemical, and phenotypic observations. Further studies combining activity-guided fractionation and direct functional interrogation of key regulatory nodes will be needed to clarify the causal basis of the proposed structure–function relationships.

*C. elegans* was used in the present study as an organism-level model to evaluate the integrated metabolic effects of the tea polysaccharide fractions. Several pathways relevant to lipid homeostasis, including insulin/IGF and AMPK signalling, are evolutionarily conserved in *C. elegans*, while its transparent body and short life cycle enable lipid accumulation, locomotion, lifespan, and stress resistance to be assessed within the same experimental system. These features make *C. elegans* particularly suitable for comparative screening of the four polysaccharide fractions. Nevertheless, the physiological complexity of mammalian lipid metabolism cannot be fully reproduced in this model, and validation in mammalian metabolic-disease models will be required before translational relevance can be established.

## 4 Conclusion

This study systematically compared tea polysaccharide-enriched fractions obtained from Tibetan dark tea processed with or without pile-fermentation and with or without golden-flower fermentation using a 2 × 2 processing design. All four fractions were identified as acidic heteropolysaccharide-rich composite systems, but they differed markedly in yield, co-extracted component profile, apparent molecular-weight distribution, monosaccharide ratios, surface charge, thermal behaviour, and microstructure.

Among the four fractions, HFTPS showed the lowest apparent molecular weight, the highest absolute zeta potential, and the highest levels of several associated components, including polyphenols and total amino acids. It also exhibited the strongest antioxidant capacity and the highest inhibitory activities against α-glucosidase, lipoxygenase, and cholesterol esterase. In a high-glucose *C. elegans* model, tea polysaccharides alleviated lipid accumulation, improved locomotion, prolonged lifespan, and enhanced heat- and oxidative-stress resistance, with HFTPS showing the most favourable overall performance among the four fractions. These phenotypic improvements were accompanied by biochemical changes and transcriptional responses consistent with improved lipid-related and stress-response regulation.

Overall, HFTPS, obtained through sequential pile-fermentation and golden-flower fermentation, exhibited the most favourable integrated structural and functional profile among the four fractions and showed promising lipid-modulating potential. These findings provide a process-oriented basis for the further development of Tibetan dark tea polysaccharides as functional food ingredients. Because the correlation analysis was exploratory and the pathway-level interpretation relied mainly on transcriptional evidence, future work should combine activity-guided fractionation and direct functional validation to clarify the causal contributions of specific structural features and regulatory pathways. Validation in mammalian metabolic-disease models will also be required before translational or clinical relevance can be inferred.

## CRediT authorship contribution statement

**Manyou Yu:** Writing – original draft, Validation, Software. **Ana Novo Barros:** Writing – review & editing. **Irene Gouvinhas:** Writing – review & editing. **Junlin Deng:** Methodology. **Zhuoya Xiang:** Resources. **Yinghao Yuan:** Formal analysis, Data curation. **Ting Zhang:** Investigation. **Xiaobo Tang:** Conceptualization. **Chen Xia:** Supervision, Funding acquisition. **Shengting Yang:** Writing – review & editing, Supervision.

## Declaration of competing interest

The authors declare that they have no known competing financial interests or personal relationships that could have appeared to influence the work reported in this paper.

## Data Availability

Data will be made available on request.
