# Supplementary material for: Pile-fermentation and golden-flower fermentation reshape tea polysaccharides in Tibetan dark tea: structural characteristics and lipid-modulating effects in Caenorhabditis elegans
Source: Food Chem X. 2026 Aug 31;39:104390. doi: 10.1016/j.fochx.2026.104390 (PMC13563591; doi:10.1016/j.fochx.2026.104390)
Supplement: Supplementary file 1 [file mmc1.docx]

**Figure S1.** Pearson correlation heatmap of physicochemical properties, *in vitro* bioactivities, *in vivo* phenotypes, and pathway-level gene response indices of four tea polysaccharides. Color intensity represents the Pearson correlation coefficient (*r*), ranging from −1 to 1, with red and blue indicating positive and negative correlations, respectively. Asterisks indicate significant correlations (**P* < 0.05, ***P* < 0.01, ****P* < 0.001). CE, cholesterol esterase inhibitory activity; LOX, lipoxygenase inhibitory activity; NR, nuclear receptor pathway index; SREBP, sterol regulatory element-binding protein pathway index; IGF, insulin/IGF-related pathway index; AMPK, serotonin–AMPK-related pathway index. Pearson analysis was performed as a supplementary validation of the overall association pattern observed in the Spearman-based correlation analysis.

**Table S1.** Primer sequences for *C. elegans* lipid metabolism-related genes.

| **Gene** | **Forward primer (5′–3′)** | **Reverse primer (5′–3′)** |
| --- | --- | --- |
| *act-1* | CTTCCCTCTCCACCTTCCAAC | GCTGGTGGTGACGATGGTTT |
| *cpt-1* | TCGAAGAATGGACACGTCGG | CACCGCCCGTTCTCATCATA |
| *ech-1.1* | GCTGGACATAAGGGGCGTAA | TGCCGCCTCTTGGTTTACTT |
| *nhr-49* | ATGGACTACTTTCTTGATGCTTCT | CCACAAACGGCACATGGTTC |
| *acs-2* | CTCAATCCTCGTATCCCGCC | GTGGTTGGGATGTTCGAGGT |
| *sbp-1* | TTGGATTGCTCGCTGGAAGT | TCCCTTCCCAGAAAAGCGTC |
| *mdt-15* | ACGACAGCAGGAAACACTCC | TGCCTGAGCTGATGTTGGTT |
| *nhr-80* | GTTGCGCGGCATCATATCAAC | ATTAGACATCGCGTGGAGCTG |
| *fat-5* | ATCCGGTGCTGATGTTCCAG | GACTGGAATGAAGGTGGGCA |
| *fat-6* | GCCCAGAGACGCAATATCTC | CAGCAAAGAGAGCCACGTTA |
| *fat-7* | ACTTCTGTTTCCGCCGTTGA | CTTGTGGGAATGTGTGGTGG |
| *sod-3* | GAAGATCGCCACCTGTGCAA | CAAGTAGTAGGCGTGCTCCC |
| *daf-16* | ACATTGCTCGAAGTGCCGAA | GTCAGATGGTAGCGGCGAAT |
| *daf-2* | GGCAACAGCCGTGACATTTT | TTTGGGGGTTTCAGACAAGT |
| *akt-1* | AGTGCAGATGAACAAAGAGGGT | CAACTCCAACTCCCCACCAA |
| *mod-1* | ACCCCTGATTGAAGAGATGCG | AGAATTTGTCCACTGCTTCCG |
| *tph-1* | TATGGATTCGTTGTTTCAGATGGC | CCTCCATGCGATTGTCACTG |
| *aak-2* | CTCTCGCTCTTCCGCCATC | GCCGTCCGTGCTTAACAATG |

**Table S2.** Effect of tea polysaccharides on the lifespan of *C. elegans*.

|  | Mean lifespan (day) | Median lifespan (day) | Maximum lifespan (day) |
| --- | --- | --- | --- |
| NC | 13.69 ± 0.67bc | 12.83 ± 0.76c | 22.00 ± 1.73d |
| CM | 11.74 ± 0.08d | 11.33 ± 0.29d | 19.67 ± 0.58e |
| OST | 13.12 ± 0.73c | 12.67 ± 0.29c | 22.33 ± 0.58d |
| LTPS | 14.31 ± 0.76bc | 13.17 ± 0.58bc | 24.67 ± 0.58bc |
| LFTPS | 13.24 ± 0.65c | 12.67 ± 0.58c | 23.34 ± 0.58cd |
| HTPS | 15.29 ± 0.23ab | 14.17 ± 0.29ab | 26.00 ± 1.00ab |
| HFTPS | 16.39 ± 1.15a | 15.00 ± 0.50a | 27.67 ± 1.53a |

Values are mean ± SD from three independent replicate plates (30 worms per plate). Different lowercase letters within the same column indicate significant differences among groups (*P* < 0.05). Asterisks are not used in this table.

**Table S3.** Effects of tea polysaccharides on *C. elegans* lifespan under heat stress.

|  | Mean lifespan (hour) | Median lifespan (hour) | Maximum lifespan (hour) |
| --- | --- | --- | --- |
| NC | 6.42 ± 0.07b | 7.00 ± 0.00c | 11.00 ± 1.00cd |
| CM | 5.11 ± 0.57c | 5.17 ± 0.76d | 9.67 ± 0.58e |
| OST | 5.37 ± 0.29c | 5.17 ± 0.29d | 9.67 ± 0.58e |
| LTPS | 6.55 ± 0.14b | 7.50 ± 0.50bc | 11.00 ± 0.00cd |
| LFTPS | 6.24 ± 0.14b | 6.83 ± 0.29c | 10.67 ± 0.58de |
| HTPS | 7.21 ± 0.13a | 7.83 ± 0.29ab | 12.00 ± 1.00b |
| HFTPS | 7.70 ± 0.62a | 8.33 ± 0.58a | 13.67 ± 0.58a |

Values are mean ± SD from three independent replicate plates (30 worms per plate). Different lowercase letters within the same column indicate significant differences among groups (*P* < 0.05). Asterisks are not used in this table.

**Table S4**. Effects of tea polysaccharides on *C. elegans* lifespan under oxidative stress.

|  | Mean lifespan (h) | Median lifespan (h) | Maximum lifespan (h) |
| --- | --- | --- | --- |
| NC | 2.74 ± 0.11c | 2.92 ± 0.14ab | 4.67 ± 0.29b |
| CM | 2.29 ± 0.06d | 2.25 ± 0.25c | 3.67 ± 0.29c |
| OST | 2.60 ± 0.18c | 2.67 ± 0.29bc | 4.33 ± 0.29b |
| LTPS | 2.82 ± 0.15b | 3.17 ± 0.29a | 4.50 ± 0.50b |
| LFTPS | 2.68 ± 0.13c | 2.67 ± 0.29bc | 4.33 ± 0.29b |
| HTPS | 3.08 ± 0.09ab | 3.08 ± 0.1ab | 5.33 ± 0.29a |
| HFTPS | 3.23 ± 0.22a | 3.33 ± 0.29a | 5.50 ± 0.00a |

Values are mean ± SD from three independent replicate plates (30 worms per plate). Different lowercase letters within the same column indicate significant differences among groups (*P* < 0.05). Asterisks are not used in this table.
